# Identification and validation of SRY-box containing gene family member *SOX30* methylation as a prognostic and predictive biomarker in myeloid malignancies

**DOI:** 10.1186/s13148-018-0523-y

**Published:** 2018-07-05

**Authors:** Jing-dong Zhou, Yu-xin Wang, Ting-juan Zhang, Xi-xi Li, Yu Gu, Wei Zhang, Ji-chun Ma, Jiang Lin, Jun Qian

**Affiliations:** 1 Department of Hematology, Affiliated People’s Hospital of Jiangsu University, 8 Dianli Rd, 212002 Zhenjiang, People’s Republic of China; 2 The Key Lab of Precision Diagnosis and Treatment of Zhenjiang City, Zhenjiang, Jiangsu People’s Republic of China; 3 Department of Nephrology and Endocrinology, Traditional Chinese Medicine Hospital of Kunshan City, Kunshan, Jiangsu People’s Republic of China; 4 Laboratory Center, Affiliated People’s Hospital of Jiangsu University, 8 Dianli Rd., 212002 Zhenjiang, People’s Republic of China

**Keywords:** *SOX30*, Methylation, Biomarker, MDS, AML

## Abstract

**Background:**

Methylation-associated *SOX* family genes have been proved to be involved in multiple essential processes during carcinogenesis and act as potential biomarkers for cancer diagnosis, staging, prediction of prognosis, and monitoring of response to therapy. Herein, we revealed *SOX30* methylation and its clinical implication in acute myeloid leukemia (AML) and myelodysplastic syndromes (MDS).

**Results:**

In the discovery stage, we identified that *SOX30* methylation, a frequent event in AML, was negatively associated with *SOX30* expression and correlated with overall survival (OS) and leukemia-free survival (LFS) in cytogenetically normal AML among *SOX* family members from The Cancer Genome Atlas (TCGA) datasets. In the validation stage, we verified that *SOX30* methylation level was significantly higher in AML even in MDS-derived AML compared to controls, whereas *SOX30* hypermethylation was not a frequent event in MDS. *SOX30* methylation was inversely correlated with *SOX30* expression in AML patients. Survival analysis showed that *SOX30* hypermethylation was negatively associated with complete remission (CR), OS, and LFS in AML, where it only affected LFS in MDS. Notably, among MDS/AML paired patients, *SOX30* methylation level was significantly increased in AML stage than in MDS stage. In addition, *SOX30* methylation was found to be significantly decreased in AML achieved CR when compared to diagnosis time and markedly increased in relapsed AML when compared to the CR population.

**Conclusions:**

Our findings revealed that *SOX30* methylation was associated with disease progression in MDS and acted as an independent prognostic and predictive biomarker in AML.

**Electronic supplementary material:**

The online version of this article (10.1186/s13148-018-0523-y) contains supplementary material, which is available to authorized users.

## Background

Acute myeloid leukemia (AML) and myelodysplastic syndromes (MDS) are common clonal disorders in myeloid malignancies. AML is etiologically, biologically, and clinically heterogeneous disease characterized by the accumulation of excessive blasts [1], whereas MDS is characterized by ineffective hematopoiesis and has a tendency to evolve into AML [2]. Cytogenetic and molecular analyses can identify recurrent chromosomal aberrations, gene mutations, and abnormal gene expression, which are associated with MDS/AML pathogenesis, response to therapy and prognosis [3, 4]. Despite recent progresses and advancements made in the understanding of disease biology and the personalized and precision treatment regimen, clinical outcome of AML even the MDS-derived AML remains unsatisfactory [1, 2]. Thus, the identification of underlying molecular events which correlated with disease progression and prognosis could make a better understanding of cancer pathogenesis and improve treatment outcome by the use of molecular risk-adapted treatment strategies [5].

DNA methylation, a common type of epigenetic DNA modification, plays a crucial role in maintenance of genome integrity, genomic imprinting, transcriptional regulation, and developmental processes [6]. In human cancers, aberrant DNA methylation is known to contribute to various biological processes of cancer development including initiation, promotion, invasion, metastases, and chemotherapy resistance [7]. Clinically, aberrant methylation in cancer-related genes acts as potential biomarkers for diagnosis, staging, prediction of prognosis, and monitoring of response to therapy [7]. As for myeloid malignancies, studies showed that aberrant DNA methylation was a dominant mechanism in MDS progression to AML [8]. For example, our previous investigation revealed that epigenetic dysregulation of *ID4*, which exhibited anti-proliferation and pro-apoptosis effects in leukemia cells, and predicted disease progression and treatment outcome in myeloid malignancies [9].

SOX [sex-determining region Y (SRY) box-containing] genes encode transcription factors belonging to the HMG (High Mobility Group) superfamily [10]. There are, at least, 19 members (*SOX1-15*, *SOX17*, *SOX18*, *SOX21*, *SOX30*) divided into eight groups (from A to H), based on their HMG sequence identity in humans [10]. The SOX genes have emerged as modulators of canonical Wnt/β-catenin signaling and have been attributed to their properties involving in the regulation of cell differentiation, proliferation, and survival in multiple essential processes during carcinogenesis [11]. Although most *SOX* genes show a property of oncogenes in various cancers, different members of the *SOX* gene family may play distinct roles in different types of cancers including hematological malignancies: some of them show an oncogenic role contributing to cancer development, whereas others act as a tumor suppressor gene to block the growth of cancers [12]. For instance, *SOX4* overexpression resulting from C/EBPα inactivation or cooperating with CREB contributed to the development of AML [13, 14]. Man et al. reported that methylation-dependent *SOX7* was a novel tumor suppressor in AML via a negative modulatory effect on the Wnt/β-catenin pathway [15]. Moreover, *SOX12* exhibited pro-proliferative effect involved in leukemogenesis by regulating the expression of β-catenin and then interfering with TCF/Wnt pathway [16]. Our previous study also showed that reduced *SOX17* expression was associated with adverse prognosis in cytogenetically normal AML (CN-AML) [17].

In this study, we examined the methylation pattern and clinical significance of *SOX30* in AML and MDS. *SOX30* methylation was a novel biomarker associated with prognosis and disease recurrence in AML and correlated with disease evolution in MDS. The results might provide us with novel insights into the mechanisms of MDS/AML leukemogenesis.

## Methods

### Patients and samples

A total of 196 AML patients (184 de novo AML and 12 MDS-derived AML), 104 MDS patients, and 28 healthy donors were enrolled in the present study approved by the Institutional Ethics Committee of the Affiliated People’s Hospital of Jiangsu University. The diagnosis and classification of MDS and AML patients were based on the 2016 World Health Organization (WHO) criteria [18]. The main clinical and laboratory features of AML and MDS patients were presented in Tables 1 and 2. After signing the written informed consents, bone marrow (BM) was collected from all participants at diagnosed time. Moreover, BM from 49 AML patients who achieved complete remission (CR) after induction therapy and 27 relapsed AML patients were also included. BM mononuclear cells (BMMNCs) were separated by density-gradient centrifugation using Lymphocyte Separation Medium (Absin, Shanghai, China) [9].

**Table 1:** Comparison of clinical and laboratory features between *SOX30* hypermethylated and non-hypermethylated AML patients

Patient’s features	Total (*n* = 196)	Non-hypermethylated (*n* = 96)	Hypermethylated (*n* = 100)	*P* value
Sex, male/female	114/82	58/38	56/44	0.564
Median age, years (range)	57 (18–86)	52 (18–83)	59 (18–86)	0.024
Median WBC, × 10^9^/L (range)	14.35 (0.3–528.0)	11.35 (0.3–528.0)	15.75 (0.3–249.3)	0.554
Median hemoglobin, g/L (range)	77 (32–147)	75 (34–147)	78 (32–144)	0.536
Median platelets, ×10^9^/L (range)	42.5 (3–447)	43 (3–447)	42 (3–399)	0.521
Median BM blasts, % (range)	49.75 (1.0^a^–99.0)	49.5 (1.0^a^–97.5)	50.5 (5.5^a^–99.0)	0.173
FAB classifications				0.005
M0	2	0 (0%)	2 (2%)	
M1	18	11 (11%)	7 (7%)	
M2	83	35 (36%)	48 (48%)	
M3	28	22 (23%)	6 (6%)	
M4	37	17 (18%)	20 (20%)	
M5	20	10 (10%)	10 (10%)	
M6	6	1 (1%)	5 (5%)	
No data	2	0 (0%)	2 (2%)	
Karyotypes				0.020
Normal	95	39 (41%)	56 (56%)	
*t*(8;21)	14	10 (10%)	4 (4%)	
inv.(16)	2	1 (1%)	1 (1%)	
*t*(15;17)	27	21 (22%)	6 (6%)	
+ 8	6	2 (2%)	4 (4%)	
-5/5q-	1	1 (1%)	0 (0%)	
-7/7q-	2	0 (0%)	2 (2%)	
*t*(9;22)	2	1 (1%)	1 (1%)	
11q23	2	0 (0%)	2 (2%)	
Complex	17	8 (8%)	9 (9%)	
Others	16	8 (8%)	8 (8%)	
No data	12	5 (5%)	7 (7%)	
Gene mutations				
*CEBPA* (+/−)	23/137	10/68	13/69	0.656
*NPM1* (+/−)	17/143	8/70	9/73	> 0.999
*FLT3*-ITD (+/−)	15/145	5/73	10/72	0.280
*C-KIT* (+/−)	10/150	6/72	4/78	0.527
*N/K-RAS* (+/−)	15/145	7/71	8/74	> 0.999
*IDH1/2* (+/−)	10/150	2/76	8/74	0.099
*DNMT3A* (+/−)	8/152	3/75	5/77	0.720
*U2AF1* (+/−)	5/155	2/76	3/79	> 0.999
*SRSF2* (+/−)	5/155	2/76	3/79	> 0.999
CR (+/−)	75/93	47/41	28/52	0.020

*WBC* white blood cells, *BM* bone marrow, *FAB* French-American-British classification, *CR* complete remission

^a^Patients’ blasts less than 20% with *t*(15;17) cytogenetic aberrations

**Table 2:** Comparison of clinical and laboratory features between *SOX30* hypermethylated and non-hypermethylated MDS patients

Patient’s features	Total (*n* = 104)	Non-hypermethylated (*n* = 80)	Hypermethylated (*n* = 24)	*P* value
Sex (male/female)	61/43	48/32	13/11	0.642
Median age, years (range)	62 (14–86)	63.5 (14–86)	67 (28–86)	0.689
Median WBC, ×10^9^/L (range)	2.7 (0.6–82.4)	2.8 (0.6–82.4)	2.5 (1.1–44.4)	0.457
Median hemoglobin, g/L (range)	64 (26–140)	66 (36–140)	56 (26–107)	0.017
Median platelets, ×10^9^/L (range)	60 (0–1176)	60 (0–754)	50 (10–1176)	0. 503
Median BM blasts, % (range)	5.0 (0.0–19.0)	5.0 (0.0–19.0)	11.0 (0.0–18.0)	0.006
WHO classifications (2018)				0.020
MDS-SLD	10	9 (11%)	1 (4%)	
MDS-RS	7	6 (8%)	1 (4%)	
MDS-MLD	32	29 (36%)	3 (13%)	
MDS-EB1	20	16 (20%)	4 (17%)	
MDS-EB2	31	18 (23%)	13 (54%)	
MDS with isolated del(5q)	3	1 (1%)	2 (8%)	
MDS-U	1	1 (1%)	0 (0%)	
IPSS scores				0.021
Low	11	9 (11%)	2 (8%)	
Int-1	52	45 (56%)	7 (29%)	
Int-2	22	16 (20%)	6 (25%)	
High	12	5 (6%)	7 (29%)	
No data	7	5 (6%)	2 (8%)	
Gene mutations				
*CEBPA* (+/−)	3/91	3/71	0/20	> 0.999
*IDH1/2* (+/−)	3/91	3/71	0/20	> 0.999
*DNMT3A* (+/−)	3/91	3/71	0/20	> 0.999
*U2AF1* (+/−)	6/88	1/73	5/15	0.001
*SRSF2* (+/−)	5/89	4/70	1/19	> 0.999
*SF3B1* (+/−)	6/98	4/70	2/18	0.604

*WBC* white blood cells, *BM* bone marrow, *IPSS* International Prognostic Scoring System

### Treatment regimen

The treatment for MDS patients with lower IPSS scores (Low/Int-1) was symptomatic and supportive treatment with/without thalidomide/lenalidomide or EPO or cyclosporine together with ATG, whereas patients with higher IPSS scores (Int-2/High) received symptomatic and supportive treatment with/without chemotherapy included decitabine or HAG protocol (cytarabine, homoharringtonine, and granulocyte colony stimulating factor) or CAG protocol (cytarabine, aclacinomycin, and granulocyte colony stimulating factor) [19]. AML patients received chemotherapy including induction therapy and subsequent consolidation treatment. For non-M3 patients, induction therapy was daunorubicin/homoharringtonine/mitoxantrone combined with cytarabine. Subsequent consolidation treatment included high-dose cytarabine, mitoxantrone with cytarabine, and homoharringtonine combined with cytarabine. Meanwhile, for M3 patients, induction therapy was oral all-trans retinoic acid (ATRA) together with daunorubicin in combination with cytarabine. Maintenance therapy was oral mercaptopurine, oral methotrexate, and oral ATRA over 2 years [9, 20].

### Cytogenetic analysis and gene mutation detection

BM cells were harvested after 1 to 3 days of unstimulated culture in RPMI 1640 medium (BOSTER, Wuhan, China) containing 20% fetal calf serum (ExCell Bio, Shanghai, China). The metaphase cells were banded by trypsin-Giemsa technique and karyotyped according to the recommendations of the International System for Human Cytogenetic Nomenclature (ISCN). Cytogenetics for AML and MDS patients were analyzed at the new diagnosis time by conventional R-banding method and karyotype risk was classified according to what was reported previously [21]. Mutations in *NPM1*, *C-KIT*, *DNMT3A*, *N/K-RAS*, *U2AF1*, and *SRSF2* were detected by high-resolution melting analysis (HRMA) as reported previously [22–26], whereas mutations in *FLT3*-ITD and *CEBPA* were detected by DNA sequencing as reported previously [27, 28].

### RNA isolation, reverse transcription, and RQ-PCR

Total RNA was isolated by using Trizol reagent and was synthesized into cDNA through reverse transcription as reported previously [9]. The primers used for *SOX30* transcript detection were 5′-TGTCACACTTTTCCAGCCCA-3′ (forward) and 5′-TGAAATCCTGTTGGCGCTCT-3′ (reverse). Real-time quantitative PCR (RQ-PCR) was performed to detect *SOX30* transcript using SYBR Premix Ex Taq II (TaKaRa, Tokyo, Japan). RQ-PCR conditions for *SOX30* transcript level detection were 95 °C for 30 s, followed by 40 cycles at 95 °C for 5 s, 66 °C for 30 s, 72 °C for 30 s, 88 °C for 30 s (collect fluorescence), and finally followed by the melting program at 95 °C for 15 s, 60 °C for 60 s, 95 °C for 15 s, and 60 °C for 15 s. The housekeeping gene *ABL* detected by RQ-PCR using 2× SYBR Green PCR Mix (Multisciences, Hangzhou, China) was used to calculate the abundance of *SOX30* transcript. Relative *SOX30* transcript level was calculated by 2^−∆∆CT^ methods.

### DNA isolation, bisulfite modification, and RQ-MSP

Genomic DNA isolation and modification were performed as reported previously [9]. Real-time quantitative methylation-specific PCR (RQ-MSP) was applied to examine *SOX30* methylation level using AceQ qPCR SYBR Green Master Mix (Vazyme Biotech Co., Piscataway, NJ, USA) with primers reported previously [29]. RQ-MSP conditions for *SOX30* methylation level detection were 95 °C for 5 min, 40 cycles for 10 s at 95 °C, 1 min at 68 °C, 1 min at 72 °C, 80 °C for 30 s (collect fluorescence), and finally followed by the melting program at 95 °C for 15 s, 60 °C for 60 s, 95 °C for 15 s, and 60 °C for 15 s. The gene *ALU* was used to calculate the abundance of *SOX30* methylation level. Relative *SOX30* methylation level was calculated by 2^−∆∆CT^ methods.

### BSP

The primers used for *SOX30* methylation density detection were 5′-TTTTTGGGTAGTAGTTATGGAG-3′ (forward) and 5′-AACTTAACCACCCTAAAAACTC-3′ (reverse). Bisulfite sequencing PCR (BSP) was conducted using TaKaRa Taq™ Hot Start Version kit (Tokyo, Japan). BSP conditions for *SOX30* methylation density detection were 10 s at 98 °C, 40 cycles for 10 s at 98 °C, 30 s at 58 °C, 30 s at 72 °C, and followed by a final 7 min at 72 °C. Clone sequencing of BSP products was performed as described previously [9], and five/six independent clones were sequenced (BGI Tech Solutions Co., Shanghai, China).

### TCGA databases

*SOX* gene family methylation (HM450) and mRNA expression (RNA Seq V2 RSEM) data in a cohort of 200 AML patients (NEJM 2013) from The Cancer Genome Atlas (TCGA) [30] were downloaded via cBioPortal (http://www.cbioportal.org) [31, 32].

### Bioinformatics analyses

The human disease methylation database DiseaseMeth version 2.0 (http://www.bio-bigdata.com/diseasemeth/analyze.html) was used for differential methylation analysis. The Genomicscape Survival Analysis (http://genomicscape.com/microarray/survival.php) was applied to determine the impact of *SOX30* expression on survival of CN-AML patients.

### Statistical analyses

Statistical analyses were conducted using SPSS software version 20.0 and GraphPad Prism 5.0. Mann-Whitney *U* test was carried to compare the difference of continuous variables between two groups, whereas Pearson chi-square analysis/Fisher exact test was applied to compare the difference of categorical variables between two groups. Correlation analysis was performed by Spearman test. Receiver operating characteristic (ROC) curve and area under the ROC curve (AUC) were carried out to test the performance of *SOX30* methylation level in distinguishing AML patients from controls. CR was obtained after one or two courses of chemotherapy. Overall survival (OS) was measured from diagnosis to last follow-up or death from any cause. Leukemia-free survival (LFS) for MDS was calculated from diagnosis to progression to acute leukemia or the end of follow-up, whereas for AML was calculated from the day that CR was established until either relapse or death without relapse. The prognostic value of *SOX30* methylation for survival (OS and LFS) was analyzed by Kaplan-Meier analysis and Cox regression analyses (univariate and multivariate analyses). All tests were two sided, and *P* < 0.05 was defined as statistically significant.

## Results

### Identification of methylation-dependent SOX gene associated with prognosis in AML

**Fig. 1:**
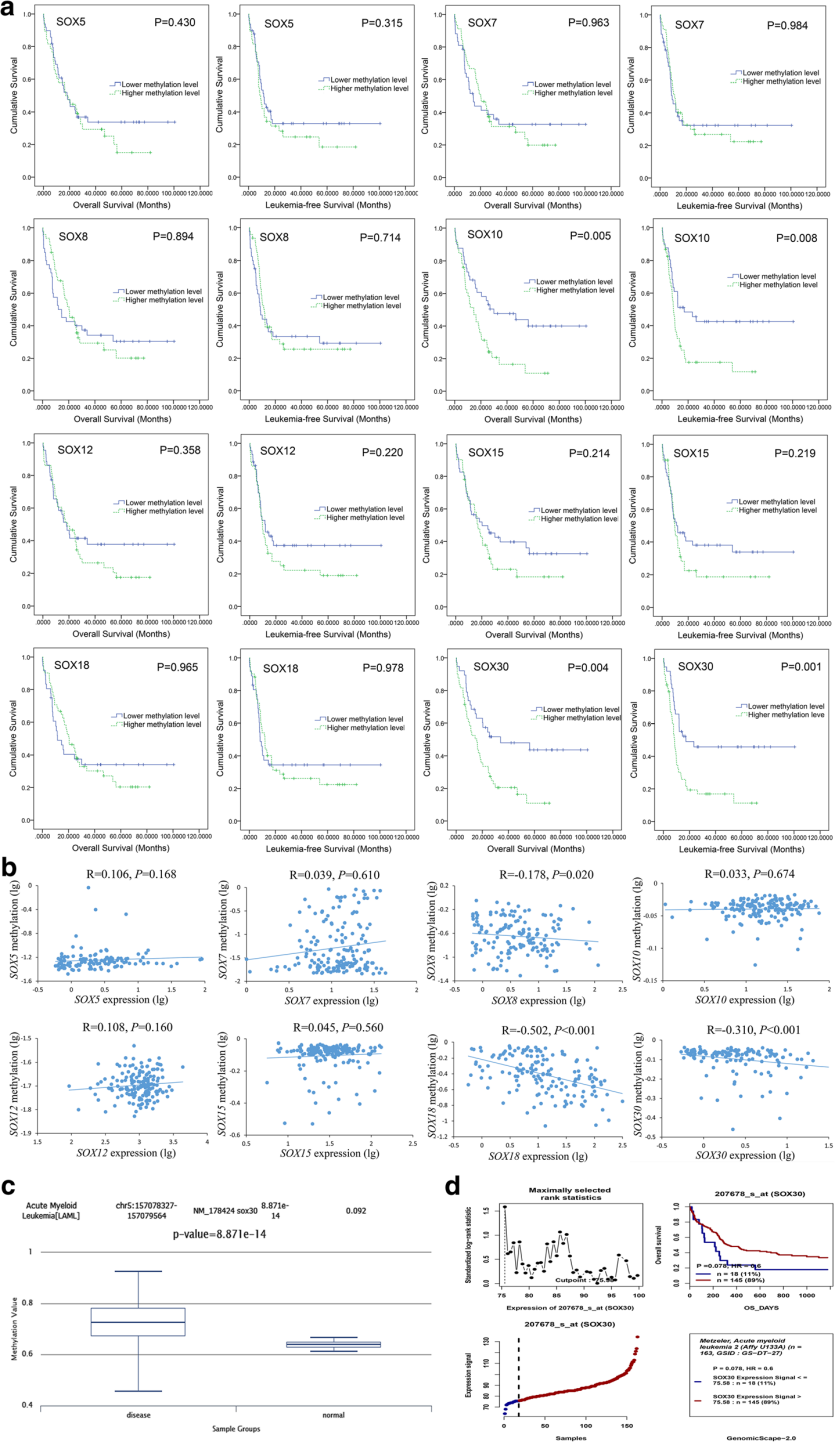
Identification of methylation-dependent *SOX* genes associated with prognosis in AML. **a** The prognostic value of *SOX* gene methylation for OS and LFS among CN-AML patients from TCGA databases. *SOX* gene methylation (HM450) data was downloaded via cBioPortal (http://www.cbioportal.org). AML patients were divided into two groups by the median methylation level of each gene respectively. **b** Correlation between *SOX* genes expression and methylation among AML patients from TCGA databases. *SOX* gene methylation (HM450) and mRNA expression (RNA Seq V2 RSEM) data was downloaded via cBioPortal (http://www.cbioportal.org). The correlation analysis was conducted by Spearman test. **c** *SOX30* methylation level in AML patients and controls obtained by bioinformatics analysis. *SOX30* promoter (CpG island) methylation level was obtained through the human disease methylation database DiseaseMeth version 2.0 (http://www.bio-bigdata.com/diseasemeth/analyze.html). **d** The prognostic value of *SOX30* expression for OS among CN-AML patients obtained by bioinformatics analysis. The effect of *SOX30* expression on prognosis was determined by the Genomicscape Survival Analysis (http://genomicscape.com/microarray/survival.php)

For initial selection of prognostic relevant methylation of *SOX* genes, we analyzed 19 members of *SOX* gene family by utilizing TCGA data. Among all *SOX* genes, methylation data was available for *SOX5*, *SOX7*, *SOX8*, *SOX10*, *SOX12*, *SOX15*, *SOX18*, and *SOX30*. To investigate their prognostic value in AML, we divided the patients into two groups by the median methylation level of each gene respectively. In whole-cohort AML patients, we did not observe the prognostic impact of *SOX* genes methylation on OS and LFS besides *SOX30* showed a trend (Additional file 1: Figure S1). However, among CN-AML, OS and LFS were adversely affected by methylation in *SOX10* and *SOX30*, but not in *SOX5*, *SOX7*, *SOX8*, *SOX12*, *SOX15*, and *SOX18* (Fig. 1a).

As is well known, DNA methylation is a major mechanism that regulates gene expression. We next analyzed the association between *SOX* gene methylation and expression in AML patients from TCGA data. Significant negative association was shown in *SOX8*, *SOX18*, and *SOX30*, but not in *SOX5*, *SOX7*, *SOX10*, *SOX12*, and *SOX15* (Fig. 1b). These data suggested methylation in *SOX8*, *SOX18*, and *SOX30* may play a major role in regulating gene expression.

Finally, we used DiseaseMeth version 2.0 (based on TCGA and Gene Expression Omnibus) to identify whether *SOX30* promoter (CpG island) was differentially methylated in AML. According to the analyses, the methylation level of *SOX30* in AML was significantly higher than normal controls (*P* < 0.001, Fig. 1c). In addition, a recent investigation reported *SOX* gene family expression in leukemia and found *SOX30* expression was significantly downregulated in AML [15]. By GenomicScape, patients with lower *SOX30* mRNA level tended to have a shorter OS time than those with higher *SOX30* mRNA level (*P* = 0.078, Fig. 1d). Taken together, we deduced that methylation-dependent *SOX30* played a crucial role in leukemogenesis.

### Validation of SOX30 hypermethylation was a frequent event and correlated with prognosis in AML

**Fig. 2:**
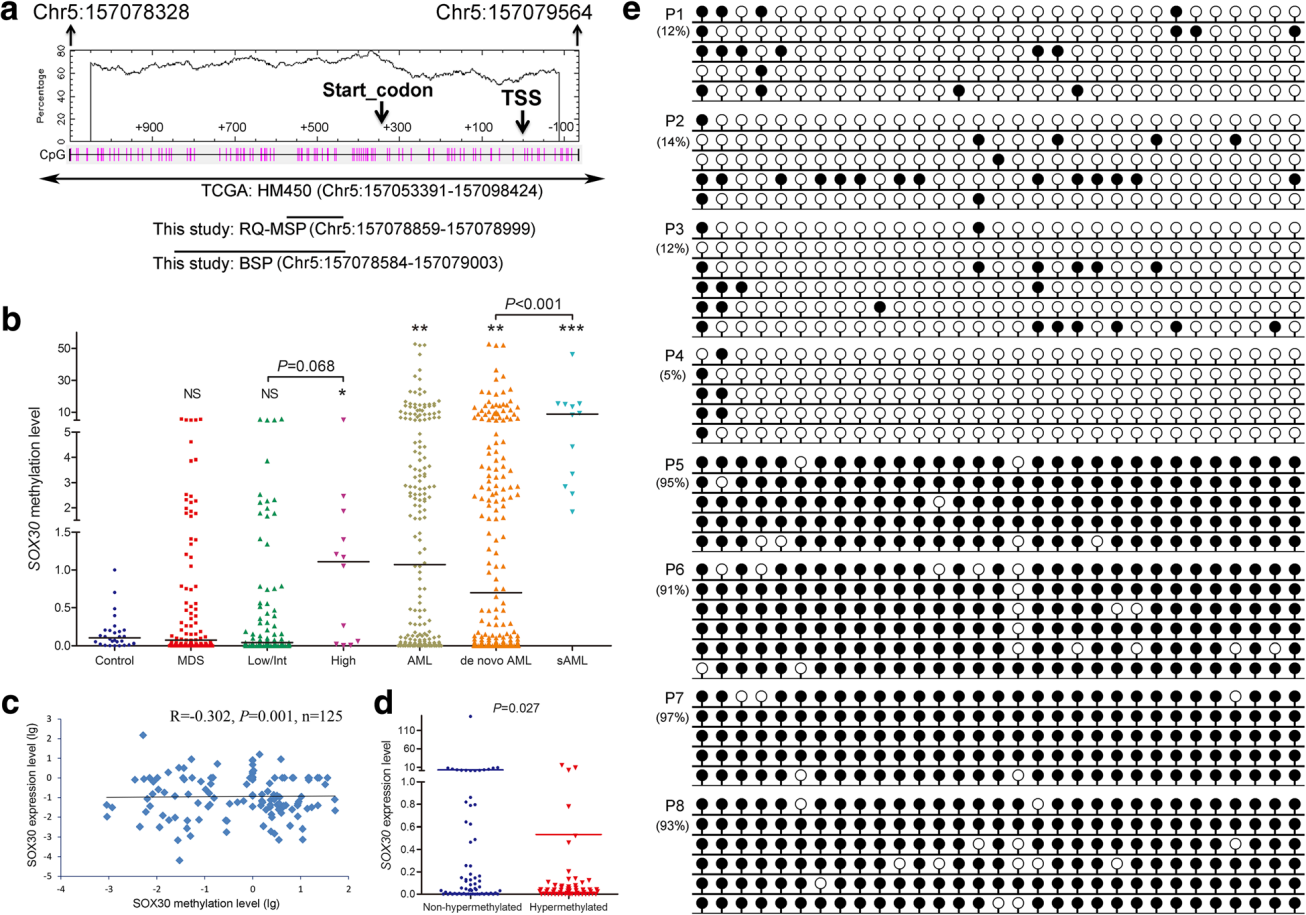
Validation of *SOX30* methylation in MDS/AML patients. **a** The genomic coordinates (GC) of *SOX30* promoter region CpG island and primer locations. The panel plots the GC content as a percentage of the total. Each vertical bar in the bottom panel represents the presence of a CpG dinucleotide. Black horizontal lines indicate regions amplified by RQ-MSP primer pairs and BSP primer pairs. CpGplot (http://emboss.bioinformatics.nl/cgi-bin/emboss/cpgplot) and Methyl Primer Express v1.0 software were used for creating the figure. TSS: transcription start site; RQ-MSP: real-time quantitative methylation-specific PCR; BSP: bisulfite sequencing PCR. **b** *SOX30* methylation level in controls and MDS/AML patients. *SOX30* methylation level was examined by RQ-MSP. Low/Int and High indicated MDS subtypes based on the classification of IPSS risks. AML included de novo AML and sAML which indicated MDS-derived AML. Each was compared to controls. NS: no significance; *: *P* < 0.05; **: *P* < 0.01; ***: *P* < 0.001. **c** Correlation between *SOX30* methylation level and expression level in AML patients. *SOX30* methylation level and expression level were examined by RQ-MSP and RQ-PCR, respectively. The correlation analysis was conducted by Spearman test. **d** *SOX30* expression level in *SOX30* hypermethylated and non-hypermethylated AML patients. *SOX30* methylation level and expression level were examined by RQ-MSP and RQ-PCR, respectively. **e** *SOX30* methylation density in controls and representative AML patients. *SOX30* methylation density was determined by BSP. P1-P2 indicated two controls selected randomly. P3-P4 represented two AML patients with lower *SOX30* methylation level. P5-P8 showed four AML patients with highest *SOX30* methylation level

We designed RQ-MSP and BSP primer sets and assays at the CpG island of *SOX30* gene promoter (Fig. 2a) to validate *SOX30* methylation in AML patients and analyzed its clinical significance. Firstly, *SOX30* methylation was examined by RQ-MSP, and AML patients had a significantly higher *SOX30* methylation level than controls (Fig. 2b). Among the tested AML patients with available RNA samples, *SOX30* expression, detected by RQ-PCR, was inversely correlated with *SOX30* methylation (*R* = − 0.302, *P* = 0.001, *n* = 125, Fig. 2c). ROC curve analysis showed that *SOX30* methylation may be acted as a potential biomarker for differentiating AML from controls with an AUC of 0.685 (95% CI 0.614–0.756, *P* = 0.002) (Additional file 1: Figure S2). We classified AML patients into two groups (hypermethylated and non-hypermethylated) based on the methylation level (1.024) at the cutoff point by ROC curve analysis (sensitivity = 51%, specificity = 100%, positive predictive value = 100%, negative predictive value = 23%). *SOX30* hypermethylated patients had significantly lower *SOX30* expression level than *SOX30* non-hypermethylated patients (*P* = 0.027, Fig. 2d). Secondly, we performed BSP in eight representative patients (two controls selected randomly, two AML patients with lowest *SOX30* methylation level, and four AML patients with highest *SOX30* methylation level) to validate the RQ-MSP results. As a result, *SOX30* methylation density was heavily correlated with *SOX30* methylation level, and the results of *SOX30* methylation density in representative AML patients were shown in Fig. 2e.

In order to analyze the clinical significance of *SOX30* methylation in AML, we compared the clinical and laboratory features between *SOX30* hypermethylated and *SOX30* non-hypermethylated groups, and results were presented in Table 1. There were no significant differences between two groups among sex, white blood cells, hemoglobin, platelets, and BM blasts. However, *SOX30* hypermethylation was correlated with higher age. Moreover, significant differences were observed between two groups in the distribution of FAB classifications and karyotype. *SOX30* hypermethylation was less frequently occurred in M3/t(15;17) subtypes. Moreover, among gene mutations, there was no significant association of *SOX30* hypermethylation with gene mutations besides *IDH1/2* mutations with a trend.

**Fig. 3:**
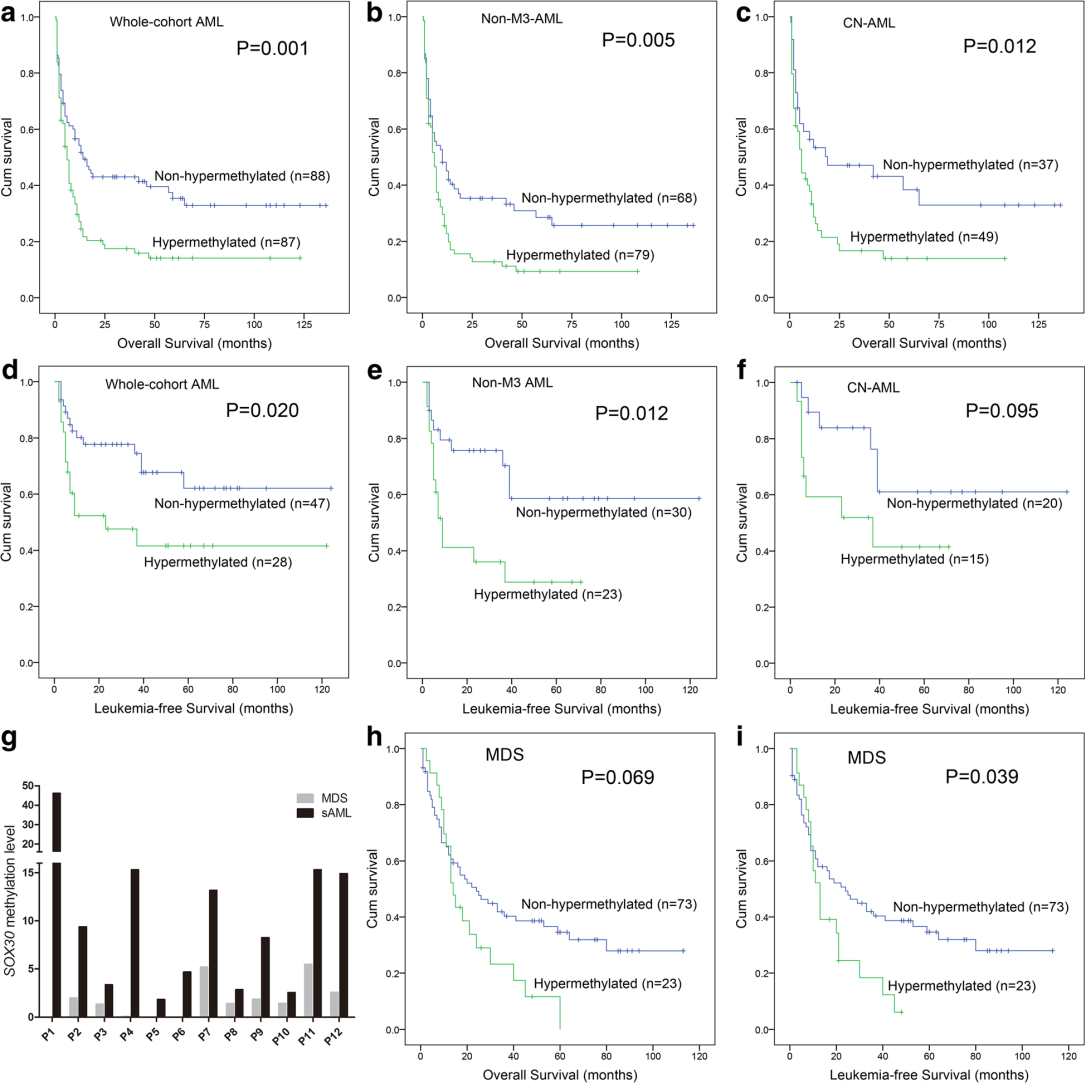
Prognostic and predictive value of *SOX30* methylation in MDS/AML patients. **a–f** The prognostic value of *SOX30* methylation for OS and LFS among AML patients. The survival analysis was performed among different subtypes of AML patients included whole-cohort AML, non-M3-AML, and CN-AML patients. **g** Dynamic changes of *SOX30* methylation level in paired MDS/sAML patients. *SOX30* methylation level was examined by RQ-MSP. sAML indicated MDS-derived AML patients. **h**, **i** The prognostic value of *SOX30* methylation for OS and LFS among MDS patients

We next determined the prognostic impact of *SOX30* methylation in AML patients. Follow-up data was available in 175 patients with a survival time ranged from 0.5 to 136 months (median 8 months). Firstly, we observed the association of *SOX30* hypermethylation with CR rate in AML patients. Among whole-cohort AML, *SOX30* hypermethylated patients showed significantly lower CR rate than *SOX30* non-hypermethylated patients (Table 1). In non-M3 AML and CN-AML, patients with *SOX30* hypermethylation tended to have lower CR rate than those with *SOX30* non-hypermethylation [44% (30/68) vs 31% (23/74), *P* = 0.121 and 54% (20/37) vs 33% (15/45), *P* = 0.074]. Secondly, by Kaplan-Meier analysis, *SOX30* hypermethylated patients had significantly shorter OS and LFS than *SOX30* non-hypermethylated patients (Fig. 3a, b). Significant difference was also observed among non-M3 and CN-AML patients (Fig. 3c–f). In addition, by Cox regression analysis, *SOX30* hypermethylation was an independently adverse prognostic biomarker for OS among whole-cohort AML, non-M3 AML, and CN-AML patients (*P* = 0.014, 0.012, and 0.054, Additional file 1: Table S1-S3).

### SOX30 hypermethylation increased the risk of leukemia transformation in MDS

Notably, for AML, *SOX30* methylation level in MDS-derived AML was significantly higher than de novo AML patients (Fig. 2a). Next, we further determined *SOX30* methylation in 12 paired patients during progression from MDS to AML. Expectedly, *SOX30* methylation level was significantly increased in AML stage than in MDS stage among all the paired patients (Fig. 3g).

### SOX30 hypermethylation was associated with higher IPSS risks and leukemia-free survival in MDS

We further investigated *SOX30* methylation in a large cohort of MDS patients. *SOX30* methylation level in MDS patients was found similar to controls (Fig. 2a). Nevertheless, *SOX30* methylation in the MDS patients with high IPSS risks were significantly higher than in controls (*P* = 0.035), and also higher than in MDS patients with low/Int IPSS risks (*P* = 0.068) (Fig. 2a). We also used the same cutoff value to define *SOX30* hypermethylation and non-hypermethylation in MDS patients. The comparison of clinical and laboratory features between hypermethylated and non-hypermethylated MDS patients was shown in Table 2. There were no significant differences between two groups among sex, age, white blood cells, and platelets. However, *SOX30* hypermethylation was associated with lower hemoglobin and higher BM blasts. Moreover, significant differences were observed between two groups in the distribution of WHO classifications and IPSS scores. *SOX30* hypermethylation was associated with MDS higher IPSS risks (Int-2/High) and WHO classifications (MDS-EB2). In addition, among gene mutations, we observed the association of *SOX30* hypermethylation with *U2AF1* mutation.

Prognostic impact of *SOX30* methylation in MDS patients was performed in 96 patients with available follow-up data (range 1–113 months, median 19 months). Kaplan-Meier analysis showed that *SOX30* hypermethylated patients had a tendency of shorter OS (Fig. 3h) and significantly shorter LFS (Fig. 3i) than *SOX30* non-hypermethylated patients. However, Cox regression analysis showed that *SOX30* hypermethylation may act as an independently adverse prognostic biomarker for LFS in MDS patients (*P* = 0.102, Additional file 1: Table S4).

### SOX30 methylation was a predictive biomarker in monitoring disease recurrence in AML

**Fig. 4:**
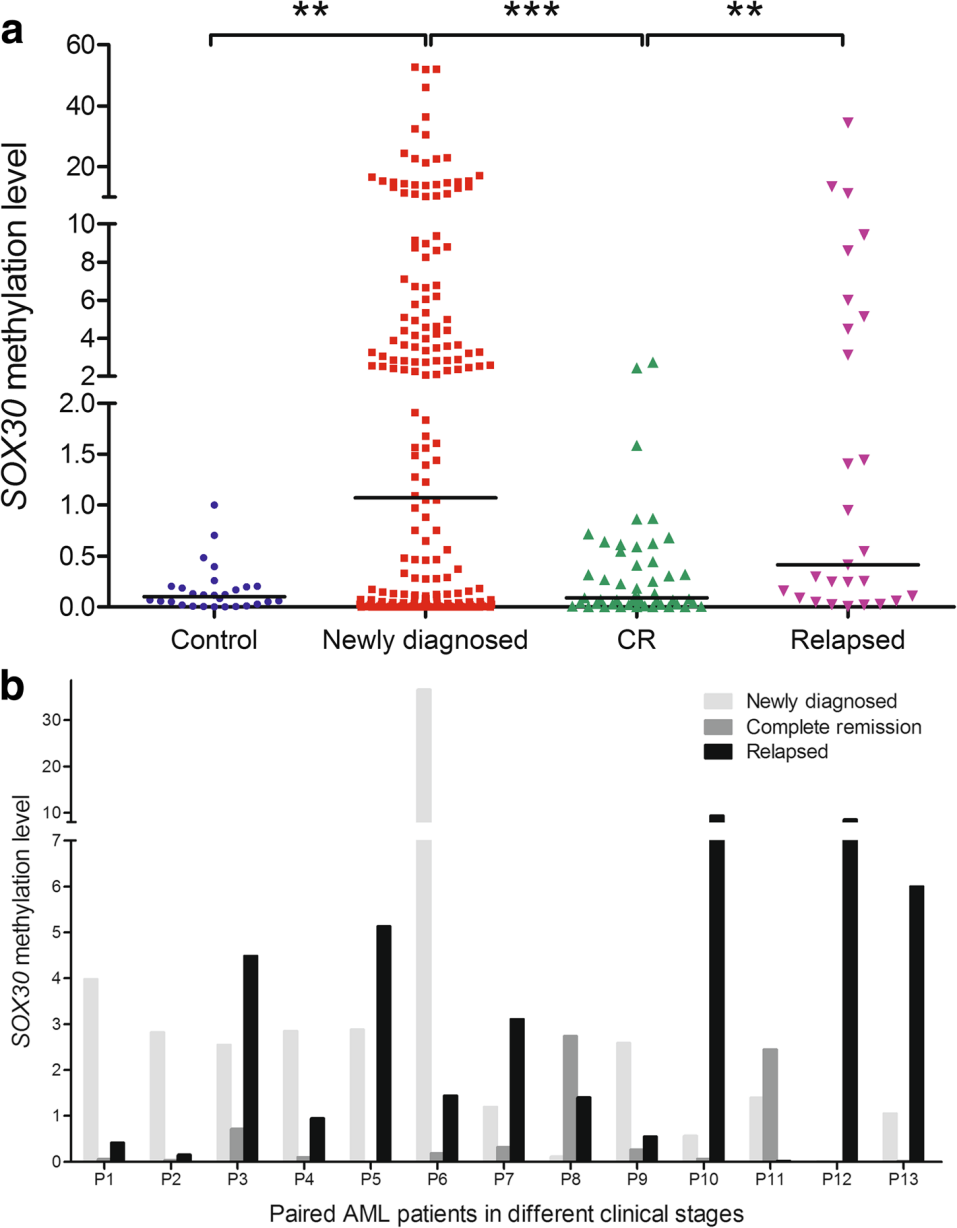
*SOX30* methylation as a predictive biomarker in monitoring disease recurrence in AML. **a** *SOX30* methylation level in AML patients of different clinical stages. *SOX30* methylation level was examined by RQ-MSP. **: *P* < 0.01; ***: *P* < 0.001. **b** Dynamic changes of *SOX30* methylation level in paired patients of different clinical stages when compared individually

To investigate whether *SOX30* methylation was a potential biomarker in the surveillance of AML, we examined *SOX30* methylation level in different clinical stages of AML patients (49 patients achieved CR and 27 relapsed patients). Significantly, *SOX30* methylation level in CR stage was significantly decreased than in diagnosis time and was markedly increased in relapsed population when compared to CR stage (Fig. 4a). Moreover, the dynamic changes of *SOX30* methylation level in 13 paired patients were also shown in Fig. 4b.

## Discussion

*SOX30*, a member of the *SOX* family, encodes a sequence-specific transcription factor that plays a vital role in gonadal differentiation and development. In species of mouse and human, *SOX30* is considered to be closely related to spermatogonial differentiation and spermatogenesis [33]. *SOX30* was reported to be highly expressed in male germ cells, human oocytes, or more differentiated cells [34]. Recently, *SOX30* has been validated to be a diagnostic, prognostic, and functional factor in several solid cancers. Han et al. demonstrated that *SOX30* was epigenetically downregulated by promoter methylation and functioned as a novel tumor suppressor partly by transcriptional activating p53 in lung cancer [29]. *SOX30* could also inhibited tumor metastasis through attenuating Wnt signaling via the regulation of β-Catenin in a transcriptional and posttranslational manner in lung cancer [35]. Furthermore, the expression of *SOX30* was verified to be closely associated with clinical outcomes in lung cancer patients [36]. Also, Guo et al. showed the anti-proliferation effect of *SOX30* overexpression in colon cancer [37]. These results indicated a non-negligible role of *SOX30* that played in the development of cancer.

In this study, we first identified *SOX30* methylation in AML from TCGA datasets and further confirmed that *SOX30* methylation were a frequent event in AML patients. In clinics of AML, *SOX30* methylation was found to be associated with older age and less frequently in FAB-M3/t(15;17), which may be caused by disease entity (less blasts in BM). Notably, *SOX30* methylation seemed to be associated with *IDH1/2* mutations despite that the *P* did not attach statistical significance. As is well known, cancer-associated *IDH* mutations are characterized by neomorphic enzyme activity and resultant 2-hydroxyglutarate production [38] and also contributed to 5-hydroxymethylcytosine depletion in cancer cells [39, 40]. Mutational and epigenetic profiling of a large cohort of AML patients revealed that *IDH1/2*-mutant AMLs displayed global DNA hypermethylation and a specific hypermethylation signature [38]. These indicated that *SOX30* methylation during leukemogenesis may be caused by *IDH1/2* mutations. For MDS, we did not observe the significant association of *SOX30* methylation with *IDH1/2* mutations and older age, which may be caused by limited cases in MDS. However, we found the association of *SOX30* methylation with *U2AF1* mutations. *U2AF1* mutations altered sequence specificity of pre-mRNA binding and splicing and was an important feature of the pathogenesis of MDS and related myeloid neoplasms [41]. Studies showed that *U2AF1* mutations caused differential splicing of hundreds of genes, affecting biological pathways such as DNA methylation (*DNMT3B*) [42]. However, the potential molecular mechanism between *U2AF1* mutation and *SOX30* methylation needs further studies. Importantly, *SOX30* methylation was associated with higher blasts, high-risk MDS, and shorter LFS time. Moreover, *SOX30* methylation showed a higher methylation level in MDS-derived AML compared to de novo AML, and the detection of *SOX30* methylation in 12 paired MDS/sAML patients showed that *SOX30* methylation level was significantly increased in AML stage than in MDS stage. These results suggested *SOX30* methylation might play a crucial role in MDS progression. By gene array technologies, Jiang et al. demonstrated that aberrant DNA methylation, more frequently than chromosome aberrations, was the dominant mechanism for tumor suppressor gene silencing and clonal variation in MDS evolution to AML [8]. However, it was the first time to report *SOX30* methylation in myeloid malignancies, whether *SOX30* functioned as a progression-related driver in MDS needed further studies.

Epigenetic modifications not only played crucial roles in cancer biology but also acted as biomarkers for cancer diagnosis and prognosis especially in blood cancer. Our previous study showed that the long non-coding RNA *H19* overexpression promoted leukemogenesis and predicted unfavorable prognosis in AML [43]. Moreover, besides mutation, dysregulation of *CEBPA* caused by its methylation was also regarded as a prognostic biomarker to guide treatment plan for AML patients [44]. *SOX30* as a prognostic biomarker has been reported in lung cancer [36]. From our study, although *SOX30* methylation was not an independent indicator in MDS, we revealed that *SOX30* methylation could act as a promising biomarker in AML. Firstly, *SOX30* hypermethylation was associated with lower CR rate, which indicated *SOX30* methylation was associated with chemotherapy response in AML. Secondly, *SOX30* methylation was associated with shorter LFS/OS and acted as independent prognostic factor in AML. Lastly, the dynamic changes of *SOX30* methylation in the surveillance of AML showed it could act as a predictor in monitoring disease recurrence. All these results suggested that determination of the *SOX30* methylation may be useful to predict long-term survival and to guide post-remission therapy in MDS and AML. Interestingly, Božić et al. also used DNA methylation profiles of AML patients from TCGA and identified a CpG site in complement component 1 subcomponent R (C1R) as best suited biomarker to further complement risk assessment in AML [45]. Obviously, prospective studies and integrative analysis are needed before we can routinely use the promising biomarkers for risk stratification and planning therapy in MDS and AML.

## Conclusions

Taken together, *SOX30* methylation was associated with disease progression in MDS and acted as an independent prognostic and predictive biomarker in AML.

## Additional file

Additional file 1: **Figure S1.** The prognostic value of *SOX* genes methylation for OS and LFS among whole-cohort AML patients from TCGA databases. *SOX* genes methylation (HM450) data was downloaded via cBioPortal (http://www.cbioportal.org). AML patients were divided into two groups by the median methylation level of each gene respectively. **Figure S2.** ROC curve analysis of SOX30 methylation for discriminating AML patients form controls. **Table S1.** Univariate and multivariate analyses of prognostic factors for overall survival in AML patients. **Table S2.** Univariate and multivariate analyses of prognostic factors for overall survival in non-M3 AML patients. **Table S3.** Univariate and multivariate analyses of prognostic factors for overall survival in CN-AML patients. **Table S4.** Univariate and multivariate analyses of prognostic factors for overall survival and leukemia free survival in MDS patients. (DOCX 660 kb)

## Abbreviations

AML: Acute myeloid leukemia
ATRA: All-trans retinoic acid
AUC: Area under the ROC curve
BM: Bone marrow
BMMNCs: BM mononuclear cells
BSP: Bisulfite sequencing PCR
CN-AML: Cytogenetically normal AML
CR: Complete remission
GEO: Gene Expression Omnibus
HRMA: High-resolution melting analysis
ISCN: International System for Human Cytogenetic Nomenclature
LFS: Leukemia-free survival
MDS: Myelodysplastic syndromes
OS: Overall survival
ROC: Receiver operating characteristic
RQ-MSP: Real-time quantitative methylation-specific PCR
RQ-PCR: Real-time quantitative PCR
TCGA: The Cancer Genome Atlas
