# Supplementary material for: Identification and validation of SRY-box containing gene family member SOX30 methylation as a prognostic and predictive biomarker in myeloid malignancies
Source: Clin Epigenetics. 2018 Jul 5;10:92. doi: 10.1186/s13148-018-0523-y (PMC6034269; doi:10.1186/s13148-018-0523-y)
Supplement: Supplementary file 1 — Figure S1. The prognostic value of SOX genes methylation for OS and LFS among whole-cohort AML patients from TCGA databases. SOX genes methylation (HM450) data was downloaded via cBioPortal (http://www.cbioportal.org). AML patients were divided into two groups by the median methylation level of each gene respectively. Figure S2. ROC curve analysis of SOX30 methylation for discriminating AML patients form controls. Table S1. Univariate and multivariate analyses of prognostic factors for overall survival in AML patients. Table S2. Univariate and multivariate analyses of prognostic factors for overall survival in non-M3 AML patients. Table S3. Univariate and multivariate analyses of prognostic factors for overall survival in CN-AML patients. Table S4. Univariate and multivariate analyses of prognostic factors for overall survival and leukemia free survival in MDS patients. (DOCX 660 kb) [file 13148_2018_523_MOESM1_ESM.docx]

**Figure S1. The prognostic value of *SOX* genes methylation for OS and LFS among whole-cohort AML patients from TCGA databases.** *SOX* genes methylation (HM450) data was downloaded via cBioPortal (http://www.cbioportal.org). AML patients were divided into two groups by the median methylation level of each gene respectively.

**
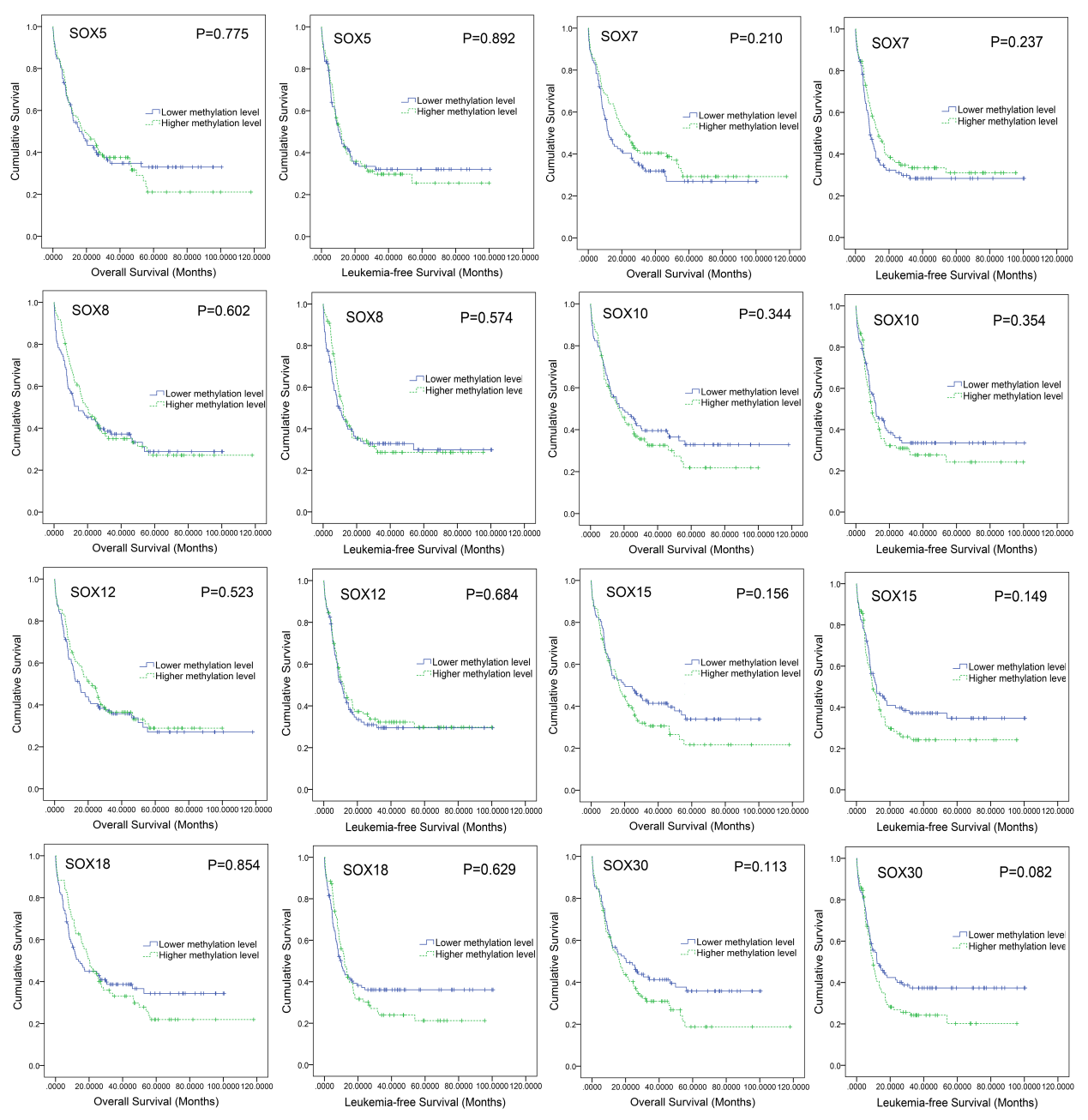
**

**Figure S2.** **ROC curve analysis of SOX30 methylation for discriminating AML patients form controls.**

**
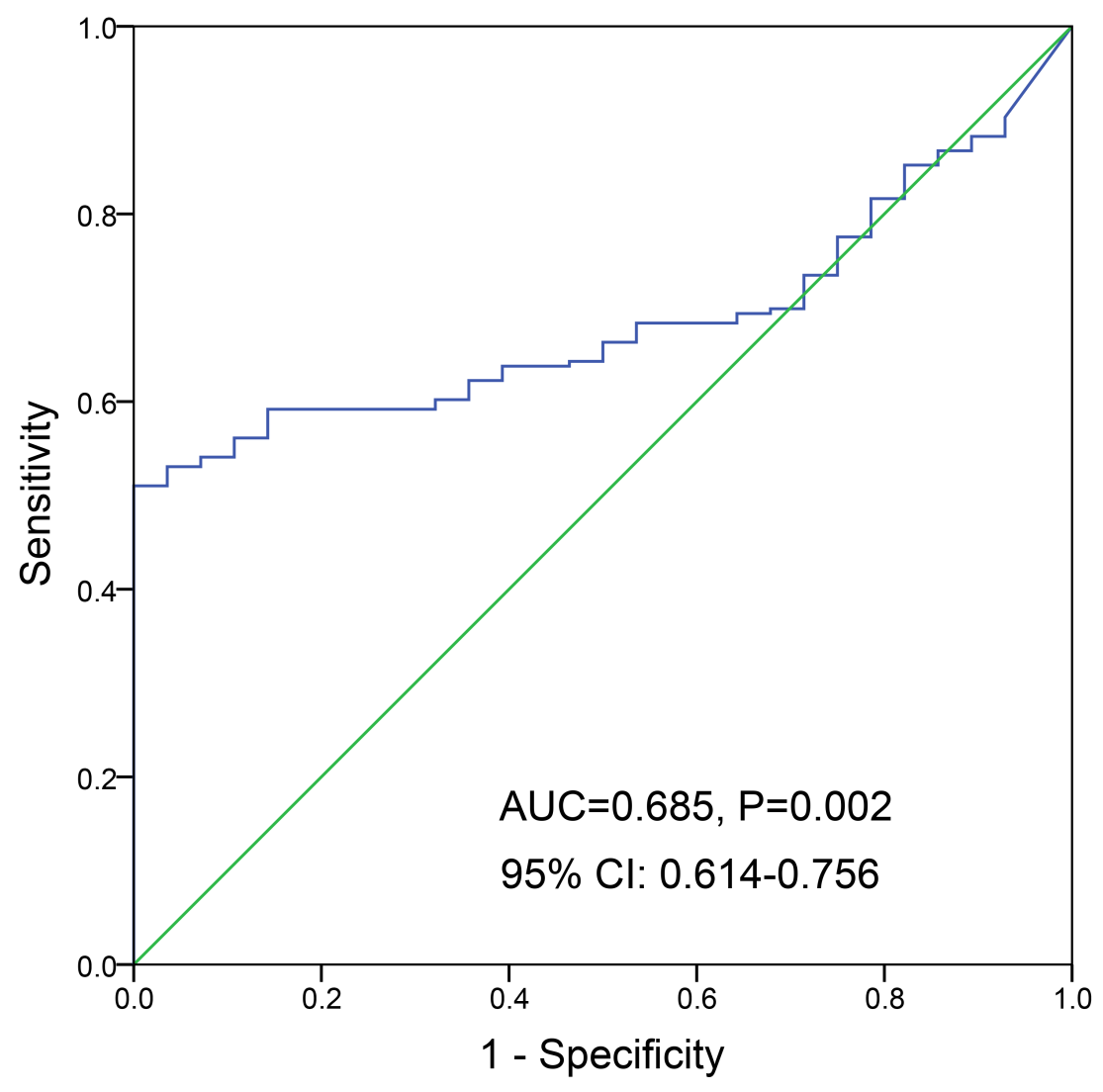
**

**Table S1. Univariate and multivariate analyses of prognostic factors for overall survival in AML patients**

| Variables | Univariate analysis | | Multivariate analysis | |
| --- | --- | --- | --- | --- |
|  | hazard ratio (95% CI) | *P* value | hazard ratio (95% CI) | *P* value |
| *SOX30* methylation | 1.812 (1.265-2.595) | 0.001 | 1.659 (1.106-2.488) | 0.014 |
| Age | 2.966 (2.067-4.256) | <0.001 | 2.160 (1.444-3.232) | <0.001 |
| WBC | 2.430 (1.690-3.493) | <0.001 | 1.767 (1.174-2.657) | 0.006 |
| Karyotype | 1.761 (1.421-2.182) | <0.001 | 1.550 (1.160-2.071) | 0.003 |
| *CEBPA* mutations | 1.157 (0.617-2.168) | 0.649 |  |  |
| *NPM1* mutations | 0.843 (0.424-1.675) | 0.625 |  |  |
| *FLT3*-ITD mutations | 0.786 (0.364-1.694) | 0.538 |  |  |
| *C-KIT* mutations | 0.853 (0.347-2.097) | 0.729 |  |  |
| *N/K-RAS* mutations | 1.158 (0.583-2.302) | 0.675 |  |  |
| *DNMT3A* mutations | 0.961 (0.391-2.365) | 0. 931 |  |  |
| *U2AF1* mutations | 3.301 (1.317-8.278) | 0.011 | 2.900 (1.147-7.333) | 0.024 |
| *IDH1/2* mutations | 1.930 (0.933-3.955) | 0.076 | 1.709 (0.765-3.815) | 0.191 |
| *SRSF2* mutations | 2.420 (0.974-6.009) | 0.057 | 1.559 (0.613-3.964) | 0.351 |

Variables including *SOX30* methylation (hypermethylation vs. non-hypermethylation), age (≤60 vs. >60 years), WBC (≥30×10^9^ vs. <30×10^9^ /L), karyotype (favorable vs. intermediate vs. poor), and gene mutations (mutant vs. wild-type). Multivariate analysis includes variables with *P*<0.200 in univariate analysis.

**Table S2. Univariate and multivariate analyses of prognostic factors for overall survival in non-M3 AML patients**

| Variables | Univariate analysis | | Multivariate analysis | |
| --- | --- | --- | --- | --- |
|  | hazard ratio (95% CI) | *P* value | hazard ratio (95% CI) | *P* value |
| *SOX30* methylation | 1.671 (1.147-2.434) | 0.008 | 1.725 (1.127-2.641) | 0.012 |
| Age | 2.366 (1.631-3.432) | <0.001 | 2.072 (1.365-3.144) | 0.001 |
| WBC | 1.975 (1.356-2.877) | <0.001 | 1.612 (1.058-2.455) | 0.026 |
| Karyotype | 1.536 (1.183-1.996) | 0.001 | 1.682 (1.197-2.364) | 0.003 |
| *CEBPA* mutations | 0.988 (0.526-1.857) | 0.970 |  |  |
| *NPM1* mutations | 0.819 (0.410-1.633) | 0.570 |  |  |
| *FLT3*-ITD mutations | 0.775 (0.358-1.675) | 0.516 |  |  |
| *C-KIT* mutations | 0.708 (0.260-1.927) | 0.499 |  |  |
| *N/K-RAS* mutations | 0.980 (0.491-1.954) | 0.954 |  |  |
| *DNMT3A* mutations | 0.816 (0.331-2.105) | 0.660 |  |  |
| *U2AF1* mutations | 3.076 (1.223-7.736) | 0.017 | 2.998 (1.181-7.606) | 0.021 |
| *IDH1/2* mutations | 1.676 (0.807-3.482) | 0.166 | 1.711 (0.762-3.842) | 0.193 |
| *SRSF2* mutations | 2.136 (0.859-5.310) | 0.103 | 1.604 (0.631-4.081) | 0.321 |

Variables including *SOX30* methylation (hypermethylation vs. non-hypermethylation), age (≤60 vs. >60 years), WBC (≥30×10^9^ vs. <30×10^9^ /L), (favorable vs. intermediate vs. poor), and gene mutations (mutant vs. wild-type). Multivariate analysis includes variables with *P*<0.200 in univariate analysis.

**Table S3. Univariate and multivariate analyses of prognostic factors for overall survival in CN-AML patients**

| Variables | Univariate analysis | | Multivariate analysis | |
| --- | --- | --- | --- | --- |
|  | hazard ratio (95% CI) | *P* value | hazard ratio (95% CI) | *P* value |
| *SOX30* methylation | 1.898 (1.120-3.217) | 0.017 | 1.880 (0.988-3.577) | 0.054 |
| Age | 2.860 (1.694-4.826) | <0.001 | 2.130 (1.150-3.942) | 0.016 |
| WBC | 2.100 (1.251-3.524) | 0.005 | 2.091 (1.132-3.862) | 0.018 |
| *CEBPA* mutations | 1.093 (0.464-2.577) | 0.839 |  |  |
| *NPM1* mutations | 0.784 (0.351-1.752) | 0.552 |  |  |
| *FLT3*-ITD mutations | 0.677 (0.268-1.709) | 0.409 |  |  |
| *C-KIT* mutations | 0.357 (0.049-2.595) | 0.309 |  |  |
| *N/K-RAS* mutations | 1.086 (0.428-2.752) | 0.862 |  |  |
| *DNMT3A* mutations | 0.991 (0.392-2.505) | 0.984 |  |  |
| *U2AF1* mutations | 2.351 (0.717-7.717) | 0.158 | 2.984 (0.869-10.246) | 0.082 |
| *IDH1/2* mutations | 1.639 (0.731-3.674) | 0.231 |  |  |
| *SRSF2* mutations | 3.272 (0.976-10.965) | 0.055 | 1.807 (0.517-6.319) | 0.354 |

Variables including *SOX30* methylation (hypermethylation vs. non-hypermethylation), age (≤60 vs. >60 years), WBC (≥30×10^9^ vs. <30×10^9^ /L), and gene mutations (mutant vs. wild-type). Multivariate analysis includes variables with *P*<0.200 in univariate analysis.

**Table S4. Univariate and multivariate analyses of prognostic factors for overall survival and leukemia free survival in MDS patients**

| Variables | Univariate analysis | | Multivariate analysis | |
| --- | --- | --- | --- | --- |
|  | hazard ratio (95% CI) | *P* value | hazard ratio (95% CI) | *P* value |
| Overall survival | | | | |
| *SOX30* methylation | 1.625 (0.952-2.755) | 0.075 | 1.365 (0.736-2.530) | 0.323 |
| IPSS scores | 1.349 (1.001-1.818) | 0.049 | 1.349 (1.001-1.818) | 0.049 |
| Leukemia free survival | | | | |
| *SOX30* methylation | 1.733 (1.011-2.972) | 0.046 | 1.308 (0.948-1.806) | 0.102 |
| IPSS scores | 1.407 (1.035-1.912) | 0.029 | 1.407 (1.035-1.912) | 0.029 |

Variables including *SOX30* methylation (hypermethylation vs. non-hypermethylation) and IPSS scores (Low vs. Int-1 vs. High vs. Int-2). Multivariate analysis includes variables with *P*<0.200 in univariate analysis.
